# Editorial: Liquid biopsy in oncology: opportunity and challenges

**DOI:** 10.3389/fmolb.2024.1466938

**Published:** 2024-07-29

**Authors:** Ashok Kumar, Rajesh Singh, Neha Arya, Smiths Lueong, Aklank Jain

**Affiliations:** ^1^ Department of Biochemistry, All India Institute of Medical Sciences (AIIMS), Bhopal, Madhya Pradesh, India; ^2^ Department of Biochemistry, Maharaja Sayajirao University of Baroda, Vadodara, India; ^3^ Department of Molecular and Human Genetics, Institute of Science, Banaras Hindu University (IoE), Varanasi, UP, India; ^4^ Department of Translational Medicine, All India Institute of Medical Sciences (AIIMS), Bhopal, Madhya Pradesh, India; ^5^ Westdeutsches Tumorzentrum Essen, Universitätsklinikum Essen, Essen, Germany; ^6^ Department of Zoology, Central University of Punjab, Bathinda, India

**Keywords:** liquid biopsy, early detection of cancer, disease prognosis, personalized medicine, circulating cell free DNA

**FIGURE 1:**
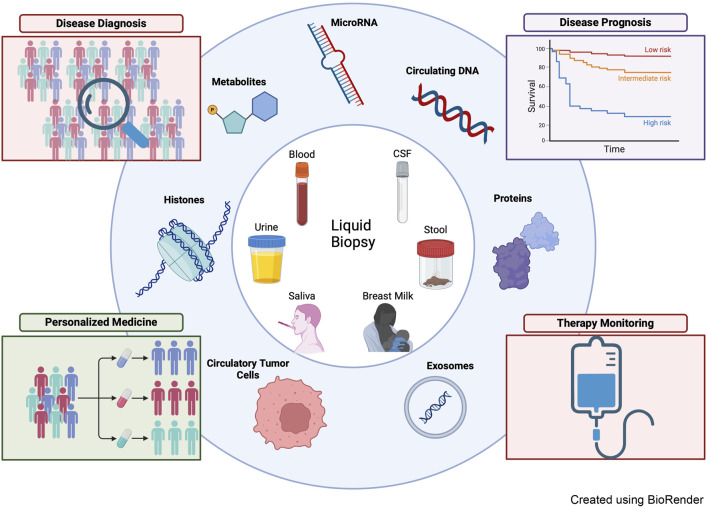
The schematic diagram depicts various biological fluids (inner circle), and cellular and molecular biomarkers (outer circle) commonly used for liquid biopsy. Rectangle boxes show the applications of liquid biopsy.

Despite significant advances in cancer diagnosis and treatment, cancer remains one of the major causes of mortality worldwide. Current challenges in cancer management include early screening and diagnosis, accurate patient stratification, appropriate treatment selection, monitoring treatment response, detecting minimal residual disease, and assessing recurrence risk. Typically, cancer diagnosis for solid tumors relies on invasive tumor biopsy, which is associated with limited access to deep-seated tumors, and intra-tumor heterogeneity. Towards this, liquid biopsy can overcome these limitations and provide a comprehensive and accurate reflection of the tumor’s molecular landscape and find application in cancer screening, classification, determining treatment options, monitoring treatment responses, and detecting minimal residual disease post-surgery. Liquid biopsy allows comprehensive monitoring of tumor progression and response to therapies by allowing multiple samplings of biological fluids. Liquid biopsy typically includes analysis of dysregulated tumor-derived exosomes, circulating cell-free DNA (cfDNA), mRNA, non-coding RNAs, and other metabolites before, during, and after treatment, thereby aiding in better diagnostic and prognostic strategies as well as personalized therapy. Figure 1 summarizes the types of body fluids that fall under the category liquid biopsy, the biomarker type and their application in cancer therapeutics. Various liquid biopsy methods have been approved by the Food and Drug Administration (FDA) including CellSearch® CTC for monitoring advanced metastatic cancers, Cobas® EGFR Mutation Test v2 for detection of EGFR mutations in non-small cell lung cancer (NSCLC) patients, and Guardant360 CDx and FoundationOne Liquid CDx for next-generation sequencing-based comprehensive genomic profiling tests for multiple solid tumors (U.S. Food and Drug Administration, 2021). However, the liquid biopsy needs more validation from the clinical counterparts. In this regard, the current Research Topic uncovers the various facets of liquid biopsy in the early detection and management of cancers.

NSCLC contributes to two-thirds of all lung cancer patients and is mostly diagnosed in advanced stages with poor survival outcomes. The mutation status of the epidermal growth factor receptor (EGFR) gene in NSCLC patients plays a crucial role in the diagnosis and follow-up of the disease and has been recommended as a part of the molecular testing-based identification under National Comprehensive Cancer Network (NCCN) guidelines for NSCLC patients (https://www.nccn.org/guidelines/Guidelines). One such study by NCCN (2024), Goksel et al. focused on NSCLC patients to determine the effectiveness of liquid biopsy in detecting various EGFR gene mutations associated with lung cancer at the time of diagnosis as well as at follow-up; the study demonstrated the superiority of liquid biopsy over tissue biopsy, as mutation detection rate was higher (32.2%) in plasma than the formalin-fixed paraffin-embedded tissues (21.9%) from lung cancer patients (Goksel et al., 2023).

Gastrointestinal (GI) cancers, including colorectal, stomach, esophageal, liver, and pancreatic, account for one-fourth of all cancers. Conventional diagnostic methods, like colonoscopy and tissue biopsy, are invasive and associated with reduced patient compliance. Mondal et al. have reviewed the recent literature on the utility of tumor-specific entities and molecular markers based on liquid biopsy with respect to GI cancers. More specifically, the authors outlined the applications of CTCs, cfDNA, ctDNA, tumor-educated platelets (TEPs), exosomes, and exosome-derived molecules in early detection, disease progression, predicting therapy response, or relapse risk in GI cancers (Mondal et al.).

The early detection and classification of pediatric brain tumors (PBT) are critical for effective treatment. Identifying and monitoring molecular markers improves pediatric tumors’ clinical management and can serve as companion diagnostic tools to magnetic resonance imaging (MRI) and histology. Towards this, cfDNA in cerebrospinal fluid (CSF) has been a focus in several studies as a minimally invasive method for diagnosing and monitoring brain tumors. However, the utility of CSF cfDNA is limited due to its quantity and integrity. Histones have been previously identified as potential diagnostic tools in various adult cancers, but not in PBT. Using ImageStream(x) technology, Buzova et al. explored the potential of cell-free histones in CSF as an alternative biomarker for the diagnosis of PBT. The study found that single histones (H2A, macroH2A1.1, macroH2A1.2, H2B, H3, H4) and histone complexes are detectable in the CSF of patients with brain tumors. These histones could provide valuable diagnostic and prognostic information, complementing existing MRI and histological analysis methods (Buzova et al.).

Prostate cancer (PCa) is one of the most common fatal malignancy. Traditional diagnostic methods, such as PSA tests and histopathology of transrectal ultrasound scan (TRUS)-guided biopsies, have limitations in specificity and risk of infection at puncture sites. Extracellular vesicles (EVs) in body fluids, have emerged as promising non-invasive tools for cancer diagnostics. Brokāne et al. focused on identifying and validating RNA biomarkers in plasma and urine EVs for prostate cancer diagnosis and monitoring. In their study, authors have validated 15 potential RNA biomarkers, identified from previous EV RNA sequencing studies from PCa patients before and after radical prostatectomy. The diagnostic potential of these biomarkers was evaluated in a test cohort and validated in an independent cohort of plasma EVs. Authors showed the significant decrease in specific RNA biomarkers (NKX3-1, tRF-Phe-GAA-3b, tRF-Lys-CTT-5c, piR-28004, miR-375-3p) in urinary EVs after prostatectomy, suggesting their association with PCa. NKX3-1 and GLO1 mRNA biomarkers notably showed promising diagnostic potential in distinguishing PCa from BPH. Combining these biomarkers with PSA improved the diagnostic accuracy for PCa (Brokāne et al.).

A study by George et al. investigates the potential of cfDNA as a biomarker for predicting outcomes in patients with acute myeloid leukemia (AML) and acute lymphoblastic leukemia (ALL). The study aimed to establish a cfDNA ratio cut-off value for risk stratification and minimal residual disease (MRD) prediction. The ratio of cfDNA reduction from baseline to the end of induction was a strong predictor of poor outcomes in high-risk patients, regardless of MRD status. A cfDNA ratio score of 2.6 or higher predicted poor outcomes with greater accuracy than conventional MRD detection by flow cytometry. A higher cfDNA ratio at diagnosis/remission or baseline indicates poor prognosis. The study suggested that cfDNA ratio scoring could be a valuable tool for prognosis in acute leukemia patients (George et al.).

While the studies published in this Research Topic demonstrate the potential of liquid biopsy for cancer detection, the studies utilized small sample sizes; further research for validating biomarkers in more extensive and diverse cohorts is warranted. In addition, most of the studies focussed on the need to explore the combinatorial use of nucleotide, protein, and metabolite to form the basis of better diagnosis/prognosis tools. The findings support the integration of liquid biopsy into routine clinical practice for personalized cancer management. Combining liquid biopsy with other diagnostic methods may enhance cancer detection and management’s overall accuracy and reliability.
